# Integrating big data and actionable health coaching to optimize wellness

**DOI:** 10.1186/s12916-014-0238-7

**Published:** 2015-01-09

**Authors:** Leroy Hood, Jennifer C Lovejoy, Nathan D Price

**Affiliations:** Institute for Systems Biology, 401 Terry Avenue North, Seattle, WA 98109 USA; University of Washington, School of Public Health, Seattle, WA USA

**Keywords:** Wellness, Personalized medicine, Whole-genome sequencing, Health behavior change, Actionable, P4 Medicine, Systems medicine, Gut microbiome

## Abstract

The Hundred Person Wellness Project (HPWP) is a 10-month pilot study of 100 ‘well’ individuals where integrated data from whole-genome sequencing, gut microbiome, clinical laboratory tests and quantified self measures from each individual are used to provide actionable results for health coaching with the goal of optimizing wellness and minimizing disease. In a commentary in *BMC Medicine*, Diamandis argues that HPWP and similar projects will likely result in ‘unnecessary and potential harmful over-testing’. We argue that this new approach will ultimately lead to lower costs, better healthcare, innovation and economic growth. The central points of the HPWP are: 1) it is focused on optimizing wellness through longitudinal data collection, integration and mining of individual data clouds, enabling development of predictive models of wellness and disease that will reveal actionable possibilities; and 2) by extending this study to 100,000 well people, we will establish multiparameter, quantifiable wellness metrics and identify markers for wellness to early disease transitions for most common diseases, which will ultimately allow earlier disease intervention, eventually transitioning the individual early on from a disease back to a wellness trajectory.

Please see related commentary: http://dx.doi.org/10.1186/s12916-014-0239-6.

## Background

In early 2014, our team at the Institute for Systems Biology initiated a 10-month pilot study called the Hundred Person Wellness Project (HPWP). Ultimately, using lessons gleaned from this pilot study, our goal is to scale the project up to 100,000 individuals – a ‘Framingham-like’ study for the digital age. The HPWP is the first real-world test of the ‘P4 medicine’ paradigm – that is, using a systems approach to ultimately transform healthcare such that it is ‘predictive, preventive, personalized and participatory (P4) [1,2].

In the accompanying commentary, Prof. Diamandis raises concerns about projects such as the HPWP, which he equates to ‘population screening’ for disease, using examples from cancer screening to elucidate his point [3]. We fundamentally disagree with the characterization of the HPWP as population screening, as well as with several additional points Diamandis makes about potential ‘harms.’ Importantly, we believe evaluating the actual outcomes of the pilot program will be far superior to supposition of harms. The three primary goals of the HPWP are:

1. To test the P4 paradigm in practice to demonstrate its effectiveness;
2. To gather dynamic, longitudinal data to create and validate proposed wellness metrics and demonstrate that their actionability creates an opportunity for the participant to optimize their wellness and for the health system to focus on identifying early disease transitions and reversing or at least mitigating them; and
3. To develop a framework for scaling these findings and to demonstrate cost effectiveness, methodologies for application in the real world and participant-centric management (rather than just health system management) of individual health.

## Discussion

There is widespread agreement that our current system of healthcare – which is ‘disease-centric’ and reactive – is not sustainable. The costs associated with treating the rising prevalence of chronic diseases such as Type 2 diabetes, Alzheimer’s, cardiovascular disease, and cancer have been predicted to bankrupt countries unless a very different approach to healthcare is adopted. The convergence of systems approaches to disease, new measurement and visualization technologies, and new computational and mathematical tools allows for the potential of P4 medicine -- which is cost-effective and increasingly focused on wellness.

While wellness and prevention may have great conceptual appeal, there are relatively few widely-accepted, quantifiable metrics to define ‘wellness’. Many individuals who report that they feel reasonable ‘well’, may, in fact, have multiple abnormalities in biochemical markers reflecting organ and system dysfunction, nutritional status or other health risk, which we have observed repeatedly in the HPWP. Thus, there is a real need to define and systemize quantifiable wellness metrics, with longitudinal data that supports their validity and clinical usefulness. Moreover, we believe that we can eventually generate a multiparameter metric for wellness -- by employing data from individuals exhibiting wellness over an extended period of time. It will reflect both the psychological and physiological aspects of wellness, thus quantifying wellness -- a concept to date that has been defined in vague and ambiguous terms.

Until recently, the potential benefits of P4 medicine were largely hypothetical [3]. The HPWP, and its subsequent scale-up to 100,000 individuals, will provide the scientific and logistical framework to test these hypotheses in a real-world setting [1]. Although the HPWP pilot project is still ongoing, there have already been interesting discoveries, many actionable findings, a tendency for some individuals to take greater control of their own health, and early validation that the promises of P4 medicine are likely to be upheld.

In the HPWP, we are gathering data in four main areas: 1) whole genome sequencing; 2) clinical and functional laboratory testing (every three months); 3) gut microbiome (every three months); and 4) quantified self and traits (physical activity, sleep, weight, blood pressure, personality and lifestyle factors, and so on). Once the results are returned, health coaches work with participants on a monthly basis to identify priority areas for lifestyle change or make referrals to physicians if medical follow-up is warranted. In addition to the data for which we are coaching, we are measuring a variety of proteomic and metabolomics markers as part of a scientific discovery effort.

Diamandis raises concerns about a number of potential harms that can result when asymptomatic individuals are studied and characterized using a large number of biometric variables, especially genetic variables [3]. Each of these concerns is addressed briefly below in the context of the HPWP.

### Does screening asymptomatic individuals potentially cause psychological distress?

There is a common myth that if a healthy individual receives information (especially genetic information) revealing increased risk for a disease that is not currently treatable, it will lead to anxiety, depression or other psychological distress. However, a fairly large body of literature contradicts this assumption. For example, Bloss *et al*. [4] looked at the responses of more than 2,000 healthy adults to direct-to-consumer genetic testing over one year and found no overall increase in health-related anxiety, with <3% of the sample reporting any degree of test-related distress. Conversely, 62% perceived the testing to be ‘of high personal utility’. Similarly, a meta-analysis of studies where people were told their genetic risk for obesity, heart disease, depression or diabetes revealed no impact on individuals’ perceived control or ‘fatalism’ [5]. Perhaps most striking, Green and colleagues [6] analyzed the psychological impact of receiving information on personal genetic risk for Alzheimer’s disease in individuals with a family history of this currently untreatable disease. They found no difference in anxiety or depression up to one year post-testing in those who received genetic results versus those who did not, regardless of whether they had the high-risk allele or not. Thus, on the basis of current evidence, one cannot argue that a positive finding in a screening test causes psychological distress, regardless of whether or not treatment or prevention is available. This concern is particularly unlikely in the HPWP where only actionable findings are being reported.

### False positives and negatives

It is inevitable that screening thousands of data points will generate false positives, as well as false negatives, and we take this concern seriously. One reason for the necessity of conducting a project on a very large scale (that is, the 100 K project) is so that appropriate analytics and methods development can be performed to improve the reliability and reproducibility of results, reducing the problem of false positives and negatives. Another key approach that we are using in the HPWP is thoughtfully tailored messaging and communications with participants about the likelihood of false positives and negatives, so that they can make well-informed decisions. Utilization of health coaches, advised by physicians, to discuss study results and provide appropriate context about false positives and negatives, particularly for newer technologies such as gut microbiome and whole-genome sequencing, is another essential element of our approach.

### Incidental findings/indolent disease

Many experts have written about the challenge of communicating incidental findings. In the realm of genetic screening, the American College of Medical Genetics has issued a formal policy for incidental findings related to certain single-gene disorders [7]. Lautenbach *et al*. [8] provide a comprehensive overview of this area, pointing out that in many cases communication of incidental genetic findings actually leads to improved health behaviors and greater patient engagement with the medical community. Similarly, in the direct-to-consumer genetic study by Bloss *et al*. [4], 36% of participants shared their genetic results with their physician, suggesting that fears of incidental findings leading to a flurry of medically unauthorized tests or procedures are unfounded. In the context of the HPWP, it is important to note that the project’s focus on wellness means that many of the findings communicated are actionable in terms of improvements to nutrition, exercise, stress management or compliance with existing medical prescriptions. These actions are thus safe, generally low-cost and consistent with practices well known to promote overall optimization of health and wellbeing. Concerns about invasive treatments with serious side-effects or harms are less of an issue when the focus is wellness rather than disease, and where those cases that could potentially indicate disease are referred to a medical provider for actual diagnosis and follow up.

### Cost-effectiveness

One of the major concerns raised about projects like HPWP that involve large numbers of ‘-omics’ measures is the cost-effectiveness, which include the costs of the tests themselves, the potential costs of additional medical care if positive findings occur, and whether or not early intervention is actually cost-saving. Regarding the costs of the testing, there is no question that today’s costs are high. However, costs are dropping rapidly and across the board so that, in the future, the cost of doing these tests will be dramatically lower. For example, today it costs from $2,000 to $4,000 to determine a human genome sequence. In a five to eight year period it is likely this cost will be reduced to close to $100 (more than an order of magnitude decrease in cost). Likewise, we acknowledge that there are potential increased short-term costs from medical care that might be initiated due to findings in the HPWP. Given that our focus is on wellness, though, the frequency of needing additional medical follow up is fairly low. To date in our project, we are only aware of one instance where an individual required short-term medical follow-up (for an elevated serum ferritin level, which was ultimately diagnosed by the provider as hemochromatosis) apart from routine physical exams and follow ups.

### The power of integrated and longitudinal data

One of the key differentiators in projects like the HPWP from standard population screening is the integration of various types of data which provide a more comprehensive assessment and, ultimately, more quantified metrics of wellness and wellness-disease transitions. For example, the *MTHFR* gene codes for an enzyme that plays an important role in processing amino acids and chemical reactions involving the B-vitamin, folate. Individuals with certain *MTHFR* variants have significantly reduced enzyme function which can lead to excess homocysteine in the blood, a known independent risk factor for cardiovascular disease: 1) with only genetic data available, an individual could just be told whether or not they have increased risk for abnormal folate metabolism and a likely increased homocysteine level; or 2) with only laboratory data available, we could tell the individual if their homocysteine was elevated but we could not tell them why or how to address it. With two integrated data types, we could provide actionable recommendations that could include either a need for increased B-vitamin intake or no need for dietary change. This example involves only two types of data, but as the project expands and additional bioinformatics reveal linkages between genetic, laboratory, microbiome and quantified-self data, the ability to deeply tailor and personalize actionable recommendations also grows tremendously.

A second key differentiator from many population-screening endeavors is the longitudinal nature of data collection. Tracking biomarkers from multiple sources (blood, urine, stool, and so on) in relation to environmental and lifestyle factors (physical activity, weight, stress, and so on) every three to six months over many years will enable the quantification of wellness with scientifically validated metrics. Further, it will identify early transitions to disease, in many cases at a stage when lower-cost interventions may still be effective or the disease can be delayed or even prevented.

### Future direction and conclusions

The launch of the HPWP pilot study is an exciting step toward a future of optimizing wellness and providing early disease prevention – one that hopefully helps catalyze a new era of medicine that is personalized, predictive, preventive and participatory.

We disagree with Prof. Diamandis’ characterization of this project as ‘population screening’, which implies a disease focus rather than the actual wellness focus of our project. On the contrary, there are broad potential benefits to both our approach and our project goals -- including scientific discovery, healthcare cost containment, innovation, optimization of individual health and wellbeing, and democratization of healthcare -- which far outweigh any possible harms. We have shown that many of the feared effects, such as psychological distress from screening results or unnecessary medical treatments, seem unlikely based on current research.

The ongoing interaction between participants and health coach and each other through social networking and community creates relationship-based accountability -- increasing motivation for long-term lifestyle change. This is rarely, if ever, an outcome of population-based screening for disease. The immediate feedback provided through longitudinal testing multi-times per year further enhances participants’ motivation and attention to wellness and prevention. Although some biomarkers take longer to improve than others, there are enough that improve in the short-term due to modifications in diet, and exercise and reduced stress, which reinforce effective health behavior change.

As new technologies and computational approaches emerge and test costs decrease dramatically -- and the interest of the general public in playing an active role in their own health and wellness continues to increase -- a transformation in our approach to healthcare is inevitable. Equally inevitable is some degree of fear and resistance from those who are invested in maintaining the status quo. This occurs during any significant paradigm shift. We are convinced that the benefits of the P4 approach to health will continue to become clear in the coming years as our project grows in numbers and the scientific discoveries, along with individual success stories of optimizing health and preventing disease, emerge.
